# Efficient and Comprehensive Representation of Uniqueness for Next-Generation Sequencing by Minimum Unique Length Analyses

**DOI:** 10.1371/journal.pone.0053822

**Published:** 2013-01-18

**Authors:** Helena Storvall, Daniel Ramsköld, Rickard Sandberg

**Affiliations:** Ludwig Institute for Cancer Research, and Department of Cell and Molecular Biology, Karolinska Institutet, Stockholm, Sweden; Tel Aviv University, Israel

## Abstract

As next generation sequencing technologies are getting more efficient and less expensive, RNA-Seq is becoming a widely used technique for transcriptome studies. Computational analysis of RNA-Seq data often starts with the mapping of millions of short reads back to the genome or transcriptome, a process in which some reads are found to map equally well to multiple genomic locations (multimapping reads). We have developed the Minimum Unique Length Tool (MULTo), a framework for efficient and comprehensive representation of mappability information, through identification of the shortest possible length required for each genomic coordinate to become unique in the genome and transcriptome. Using the minimum unique length information, we have compared different uniqueness compensation approaches for transcript expression level quantification and demonstrate that the best compensation is achieved by discarding multimapping reads and correctly adjusting gene model lengths. We have also explored uniqueness within specific regions of the mouse genome and enhancer mapping experiments. Finally, by making MULTo available to the community we hope to facilitate the use of uniqueness compensation in RNA-Seq analysis and to eliminate the need to make additional mappability files.

## Introduction

Next-generation sequencing based methods have in the last couple of years increased enormously in usage. Common to next generation sequencing methods is the fragmentation of DNA or RNA into smaller pieces which are amplified, whereupon short reads from millions of these fragments are sequenced in parallel [1]. The length of the sequenced reads typically ranges from around 25 to 150 base pairs for most applications. The origins of the reads are then determined by mapping them back to the genome. Finding the origins is not always straightforward though, since the genome contains repetitive regions caused by transposable elements, tandem arrays and gene duplicates which may cause reads to map to more than one place in the genome. For short reads, the same sequence could also occur in several places just by chance. The mappability can to some extent be improved by performing paired-end sequencing, where two reads from each DNA or RNA fragment is sequenced – one from each end. In this case a fragment can become uniquely mapped although one read is non-uniquely mapping to a repetitive region. Depending upon application, “multimapping” reads are often excluded from analysis since their origin cannot be unambiguously determined.

When performing transcriptome sequencing, expression levels of different genes are determined by counting the number of reads mapping to the gene and normalizing this read count by the length of the gene model and the total number of mapped reads in the sample [2]. Thus, expression levels are expressed as number of reads per thousands of base pairs of gene model and million mappable reads (RPKM) that enables comparison of expression levels both between genes of different lengths and between samples of different sequence depths. However, a problem emerges when a large portion of a gene is not uniquely mappable. The read count in this region will be deceptively low if only the uniquely mapping reads are counted, and as a result the calculated expression level will not reflect the true expression level.

Previous efforts to compensate for the lack of mappability have normalized reads counts with the number of uniquely mappable positions [3]–[5] instead of full transcript length. These studies have often stored whether reads of a particular length are uniquely mappable at any given genomic coordinate, e.g. encoding this uniqueness information in either chromosomal fasta files with a capital letter to indicate unique mappability, in Wig formats, of by storing the number of occurrences of the given read in the genome [6]–[9]. A major limitation with these types of files is that they need to be generated for each read length encountered, and also, compiling and storing uniqueness files for large number of read lengths requires excessive data storage [5], [6], [10]. Alternative approaches instead include multimapping reads, by assigning them to their most likely origin based on either how many *unique* reads map to the surrounding region [2] or on the total number of reads (unique and multimapping) that potentially comes from the region [11]. Assigning multimapping reads to genes based on the coverage of uniquely mapping reads could be problematic, since multimapping reads will more likely be assigned to regions of high uniqueness, and could therefore lead to biases in gene expression levels. Although these alternative methods have been described in the literature, no study to date has compared their uniqueness compensation.

In this paper we present a novel approach to efficiently and comprehensively describe mappability of a genome or transcriptome. We create a single uniqueness file per chromosome for each genome with mappability information across the full *range* of useful read lengths. The files contain values representing the minimum read length required for uniqueness at each genomic position, which enable fast and easy querying for uniqueness across arbitrary read lengths. We also developed similar uniqueness files for transcriptomes (including exon-exon junctions) to get improved mappability information for spliced mRNA. Using the uniqueness information for the transcriptome files, we compared the alternative approaches for handling multimapping reads in RNA-Seq and found that using these uniqueness files for transcript quantification results in the most effective compensation for transcripts with low uniqueness. Similar uniqueness files were also developed for bisulfite sequencing and paired-end RNA-Sequencing experiments and we explored the mappability to give guidelines for the required read length for different sequencing applications. Finally, the uniqueness data and framework developed within this study can easily be used in experiments of arbitrary read lengths and therefore facilitate the use of uniqueness compensation in RNA-Seq analyses.

## Results

### Comprehensive uniqueness representation using the minimum unique length

**Figure 1:**
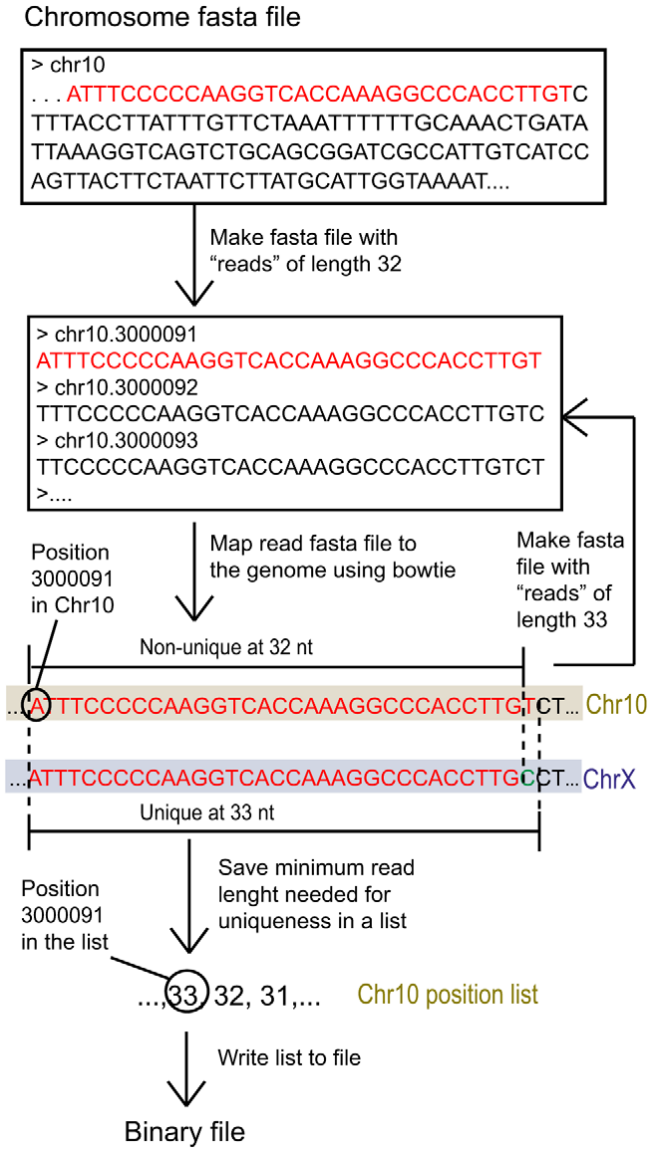
Schematic illustration MULTo file generation. (**A**) We defined the minimum unique length (MUL) of a genomic coordinate as the length of the shortest starting oligonucleotide at that coordinate that is needed to be unique. To find the MUL value, Fasta files with artificial “reads” of different lengths were iteratively created from whole chromosome fasta files and mapped to the genome using bowtie. When the minimum length needed for uniqueness was found, this value was stored in a binary file. In this example, position 3000091 was unique at 33 base pairs but not at 32, i.e. we have a MUL value of 33. (**B**) Exemplifying that MUL values can be retrieved from arbitrary regions in just a few lines of code.

We reasoned that instead of storing whether a read of predetermined length is unique at a given genomic coordinate, it would be more efficient to store the minimum length required for each genomic coordinate to be uniquely mappable, which we call the minimum unique length (MUL). To find the minimum unique length for every position of a mammalian genome, we developed an iterative re-mapping scheme as illustrated in Fig. 1 and Fig. S1. By default we queried read lengths between 20 and 255 nucleotides (nt). The upper limit is chosen to enable efficient data storage – a byte in a binary file can take on values between 0 and 255 – while the lower limit is chosen because sequence reads are seldom shorter than 20 nt. At the same time, this range covers the read lengths used in most genomic and transcriptomic studies. We created MULTo files for the mouse and human genomes (mm9 and hg19, respectively), requiring only 2.5 Gigabytes of storage (about the same size as Fasta files with uniqueness information for one read length). Since each genomic coordinate is stored as a number it makes queries at arbitrary locations and for arbitrary read lengths both simple and efficient. The MULTo package also includes a search function to easily retrieve the uniqueness for a set of genomic regions.

### Uniqueness profiles within transcribed regions

For transcriptome analyses, sequenced reads are required to be unique in both the genome and transcriptome. We extended our analyses to consider transcriptomes and to correctly handle splice junctions. In expression level quantification, reads mapping to all isoforms of a genomic locus are often being used to estimate gene expression levels, whereas reads mapping to isoform-specific regions can be used to deconvolute isoform expression levels. We created transcriptome MULTo files for both purposes. For gene expression quantification, a read was considered unique if it mapped to one or more transcripts but at the exact genomic locus (defined as having the same start or end coordinate after converting the transcript coordinates to genomic coordinates). Alternatively, we generated MULTo files with only reads that map uniquely to *one* transcript variant, with a caution that the uniqueness profiles are highly sensitive to the completeness of the transcript annotations used. These two strategies are illustrated in Fig. S2, and we used the less stringent (gene-level) files to compensate for mappability when estimating expression levels.

We also estimated uniqueness information in paired-end sequencing data, although the situation is more complex than for single-end read. In paired-end uniqueness one has to consider not only read lengths but the insert length. Moreover, the insert length for a sequencing experiment is never an absolute value but rather a normal distribution of lengths. Therefore we first simulated inserts lengths around a mean value, and then counted the proportion of uniquely mapping across the inserts, before we obtain an estimate of the proportion of unique positions within each transcript (see methods).

### Gene-level transcriptome uniqueness

**Figure 2:**
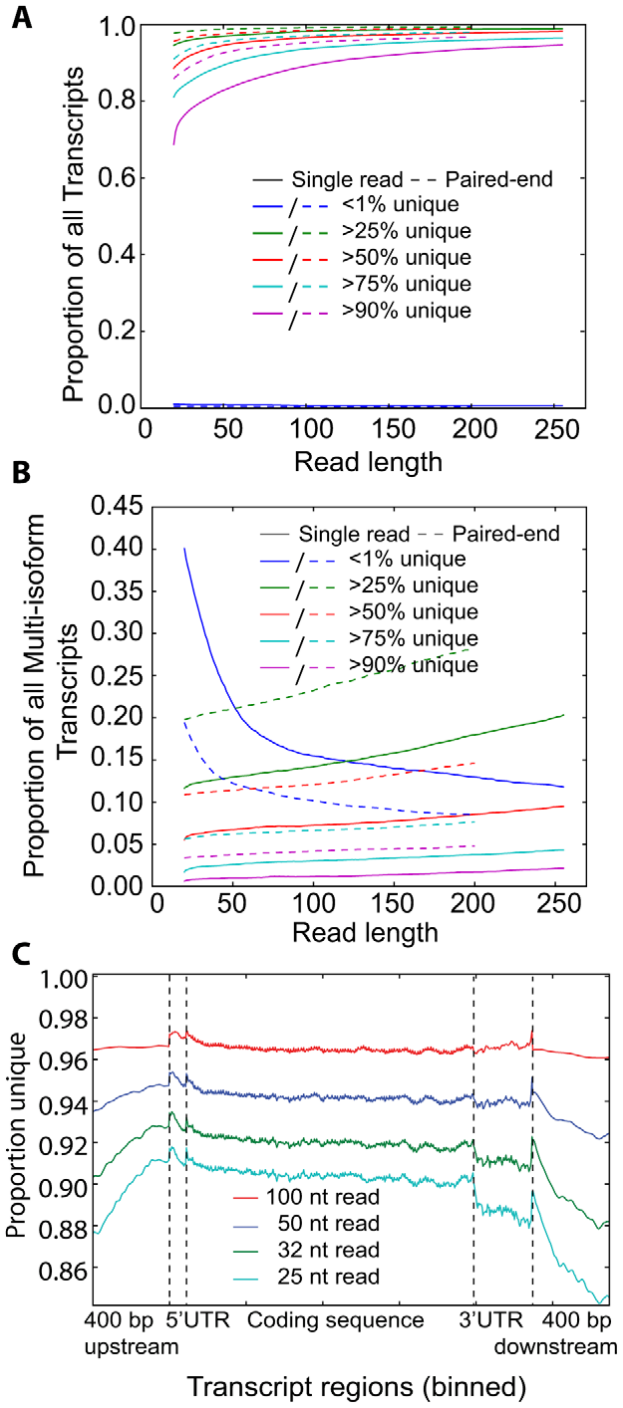
Uniqueness in the transcriptome. (**A, B**) We calculated the proportion of unique positions for each transcript, both for single reads and paired-end fragments (mean 500 nt), and then plotted how many transcripts have a certain proportion of unique positions. The y-axis represents the proportion of all transcripts that satisfies the given condition. (**A**) Gene-level uniqueness of all RefSeq transcripts. (**B**) Transcript-level uniqueness for all transcripts from multi-isoform genes. (**C**) Positional plot of the uniqueness proportion across all coding transcripts. We calculated the number of reads of a specific length that passes through each position, and determined what proportion of these were unique. Since transcripts differ in length, we binned positions together so that each region (upstream, downstream, coding sequence, 5′ and 3′UTR) had the same number of bins for each transcript. The x-axis represents coordinate bins across transcripts.

Using mouse RefSeq transcript annotations, we explored the fraction of genes with different proportions of uniquely mappable positions in both single-read and paired-end data. We found that the transcriptome effectively collapsed into two classes of gene-level uniqueness, transcripts with high or low mappability regardless of read lengths (Fig. 2a). Already at 50 nt, over 83% of the transcripts were unique in 90% or more of its mappable positions, and increasing the read length to 255 increased the fraction of transcripts to 95%. Still, a few percent of transcripts had no mappability even at 255 nt read lengths. With paired-end information we found 93% of transcripts being unique at 90% or more mappable positions for 50 nt reads and 500 nt inserts (Fig. 2a).

### Transcript-level transcriptome uniqueness

Exploring the transcript-level uniqueness for genes with two or more isoforms, we found that most transcripts had very low proportions of unique positions (Fig. 2b). At 50 nt only about 13% of all transcripts had more than 25% unique positions. Using paired-end data increased this percentage to 21% of transcripts, and at 200 nt read lengths this percentage increase further to 28%. Thus, both paired-end sequencing and longer read lengths are important for analyses of isoform-specific expression level, but still only a limited amount of fragments are directly informative. In agreement with a recent paired-end RNA-Seq study [12], we found that isoform-specific mappability increased with the insert length in paired-end sequencing libraries (Fig. S3). Finally, we examined the uniqueness across transcripts and found a fairly even uniqueness profile with lower uniqueness in 3′UTRs for shorter read lengths (Fig. 2c).

### The effect of uniqueness length compensation in RNA-Seq

Our main motivation in creating the MULTo resource was to be able to normalize expression values by the number of uniquely mappable positions, regardless of the read length sequenced in a particular experiment. We have recently published a flexible program for expression level estimations (rpkmforgenes) that only considers uniquely mapping reads and corrects the gene model length for only uniquely mappable positions [13]. We incorporated the gene-leve uniqueness files from MULTo into rpkmforgenes to allow for unique length normalization at any read length (Sandberg lab RNA-Seq tools. Available: http://sandberg.cmb.ki.se/rnaseq, Accessed Dec 12 2012).

**Figure 3:**
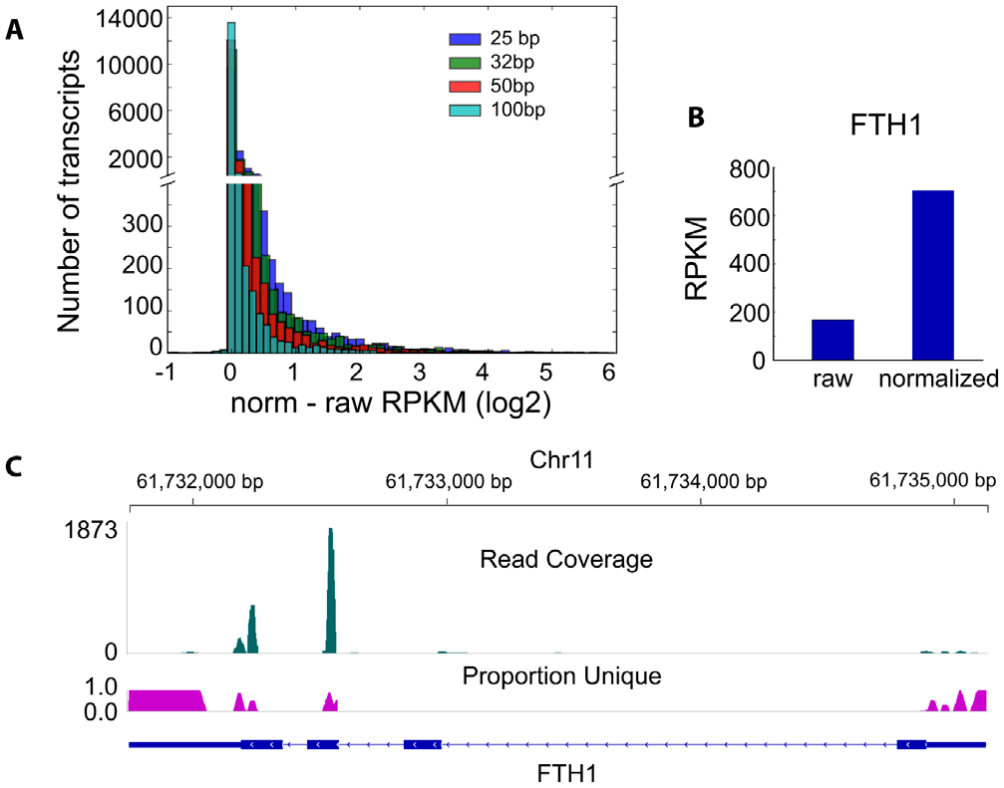
Effects of uniqueness normalization on expression level. (**A**) Histogram showing how uniqueness compensation using MULTo affects the RPKM values at different read lengths. The x-axis show the difference in gene expression between uniqueness compensated and uncompensated expression levels. (**B**) RPKM values for FTH1 before and after uniqueness normalization. (**C**) Read coverage and uniqueness profile across FTH1 for 25 nt reads. Uniqueness density was calculated as the proportion unique reads aligning to each genomic coordinate.

To study the impact of uniqueness compensation on gene expression level estimation, we compared RPKM values estimated from uniquely mapping reads using either the full transcript lengths (raw RPKM) or after correcting transcript lengths for uniquely mappable positions (norm RPKM). Naturally, the lack of proper uniqueness length compensation confounded the estimates for lower expression levels. This is apparent from Fig. 3a, where we plot differences in RPKM (norm/raw) at different read lengths. To control the comparison for only length effects, we used a large, mixed-tissue, RNA-Seq dataset of 100 bp reads and iteratively trimmed the Fastq reads from a 100 bp experiment down to the shorter lengths. While most genes were not significantly affected by the uniqueness normalization, we found 764 genes whose RPKM values increased two-fold or more after mappability compensation for 25 bp reads, and 162 genes for 100 bp reads. The overall differences in RPKM increased with shorter read lengths (since they have fewer uniquely mappable positions) and for particular genes the expression difference can be more than 10-fold. The ferritin heavy chain gene (FTH1) is an example of a gene highly dependent on uniqueness normalization for correct expression values (Fig. 3b–c). FTH1 has three paralogues with highly similar sequence, resulting in only 42% of the positions in gene being uniquely mappable at 25 base pairs. Uniqueness normalization for FTH1 in our RNA-Seq data increased the RPKM more than 4-fold (Fig. 3b), and visual inspection of the read coverage confirms only reads at uniquely mappable positions (Fig. 3c). These results show the importance of normalizing RNA-Seq data to the number of uniquely mappable positions in a gene when estimating absolute expression levels, especially when using short reads.

### Comparing uniqueness compensation methods for RNA-Seq

**Figure 4:**
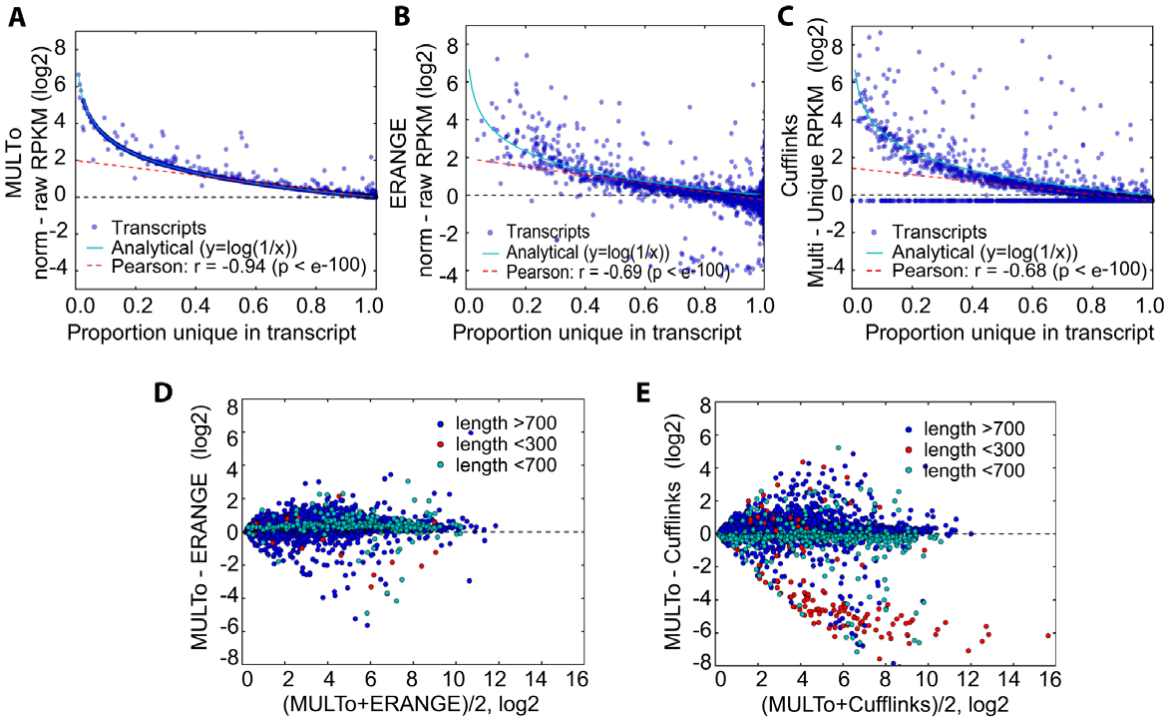
Comparison of uniqueness compensation methods for RNA-Seq. Scatter plots showing how gene expression values (RPKM) are affected by uniqueness compensation for transcripts as a function of increasing proportion unique positions. (**A**) Uniqueness compensation with MULTo corrected transcript lengths are close to optimal compensation line (y = 1/x) (**B**) ERANGE uniqueness compensation. (**C**) Cufflinks uniqueness compensation. (**D,E**) MA-plots between MUL and ERANGE uniqueness compensation (D) and between MUL and cufflinks uniqueness compensation (E) showing how gene expression differences correlate to the gene expression average. Short transcripts were colored red.

At least three different methods have been used to compensate for the lack of uniqueness in RNA-Seq analyses although no study has compared their compensation ability. We therefore sought out to compare how these methods compensate for uniqueness on the exact same RNA-Seq data. Using the MULTo files we derived the proportion of uniquely mappable positions within each transcript and we compared the effects of uniqueness compensation on transcripts as a function of the numbers of uniquely mappable positions. Accurate compensation methods should show gene expression changes that followed the expected distribution (y = 1/x, where x is the proportion uniquely mappable positions). We first evaluated uniqueness compensation by discarding multimapping reads and adjusting the gene models to only include uniquely mappable positions (using rpkmforgenes). Comparing expression levels (as RPKMs) generated with only uniquely mapping reads and full (raw) or adjusted (norm) gene model lengths revealed that essentially all genes followed the expected line (Fig. 4a), demonstrating that gene expression estimates with rpkmforgenes generate intuitive and accurate uniqueness compensation. The few points that deviated from the line were likely the results of noise in the separation of reads from multiple overlapping transcripts. Importantly, analyzing the uniqueness compensation as a function of the numbers of uniquely mappable positions within transcripts provides useful diagnostic and we next evaluate the two other methods.

In the early ERANGE method [2], raw RPKM values were first calculated based on uniquely mapping reads and full transcript length, followed by the assignment of multimapping reads to the most likely transcript of origin based on the raw RPKM. We compared those raw RPKM values to those achieved by ERANGE after multimapping correction. The comparison revealed a partial compensation for uniqueness that followed the expected distribution (y = 1/x), with a slight but widespread underestimation of expression levels for transcript with lower fractions of unique positions and a few outliers that were over-estimated (Fig. 4b). A similar approach for multimapping assignments is used in cufflinks, where they score the probability of each read to map to all its possible positions, and then calculate expression levels as the likelihood of a transcript abundance, given the set of uniquely mapping and multimapping fragments [11]. We computed expression levels in cufflinks (as FPKM using the multi-read option) on the same alignment files and compared their expression levels to the raw uncorrected levels that used only uniquely mapping reads. Again, we found more variability in the uniqueness compensation (Fig. 4c), with frequent outliers that were either overestimated and a group of genes with low mappability that failed to be compensated using the multimapping reads.

Although the computed gene expression values from these three methods were in general agreement (Fig. 4d–e), a great deal of variation was introduced by their respective methods for uniqueness compensation, since the gene expression values derived from only using uniquely mapping reads show much less variability (Fig. S4a–b). In these comparisons we noted that Cufflinks tends to overestimate the expression levels of short transcripts (in particular those below 300 bp) (Fig. 4e), but this disconcordance was not due to mappability, since Cufflinks overestimated this class of transcripts using uniquely mapping reads alone (Fig. S4c). Finally, the expression values in Cufflinks depend upon the number of multimapping reads allowed in the initial TopHat mapping. Some transcripts vary ten-folds in expression from TopHat mappings that allowed for 20 or 255 multimapping reads (Fig. S4d).

### Mappability profiles of genes and regulatory regions

**Figure 5:**
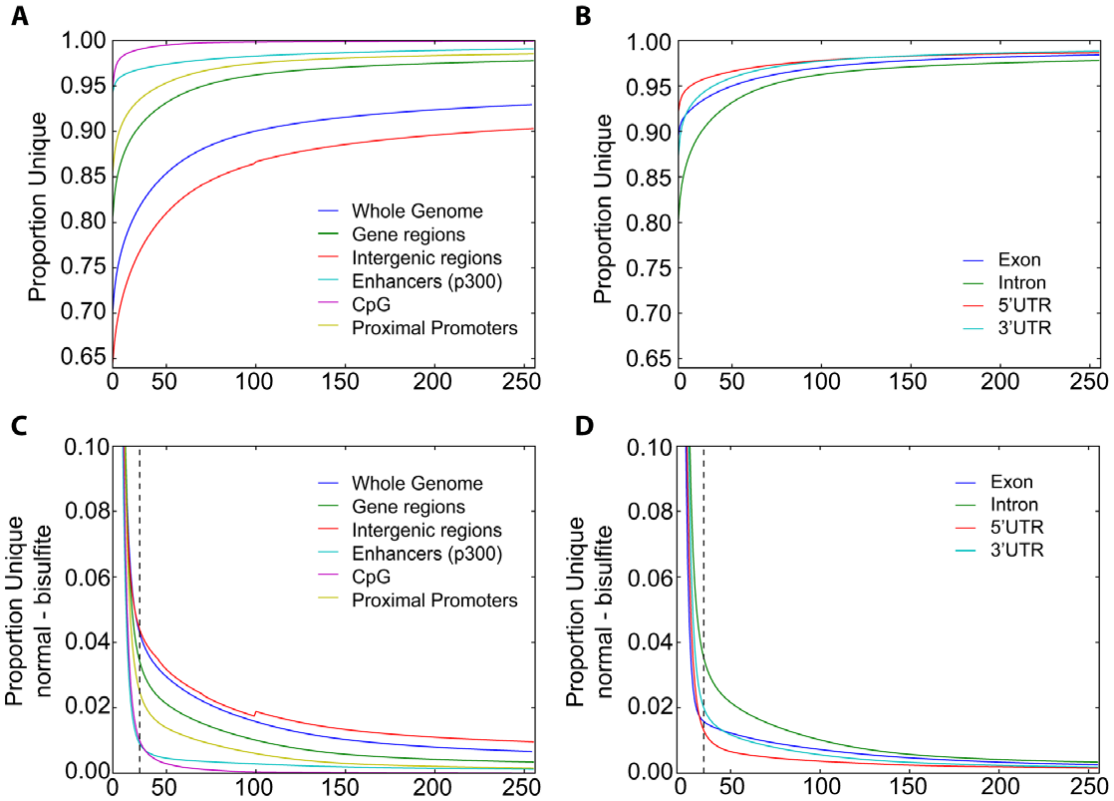
Uniqueness profiles within genomic regions. The proportions of unique positions within different regions were calculated for read lengths in the range 20–255 nts. (**A**) Proportion unique positions in whole genome, within RefSeq genes, intergenic regions, known p300 binding sites, proximal promoters and CpG islands. (**B**) Proportion unique positions within different parts of genes; exons, introns and UTRs. (**C,D**) Difference in proportion unique positions between the regular and bisulfite converted genome. The y-axis in (C) and (D) represents the uniqueness proportion in bisulfite genome subtracted from that in the regular genome. The vertical dashed line marks 35 nucleotide reads.

The mappability of the genome is also relevant for analysis of other types of sequencing data, including ChIP-Seq, (Chromatin Immunoprecipation coupled with sequencing), DNAse hypersensitive site and bisulfite sequencing. The ability to distinguish ChIP-Seq peaks over background can be improved by mappability normalization [9]. We investigated the patterns within regulatory regions and compared those with annotated genes and intergenic regions. Proximal promoters (here defined as the 1 kb upstream of annotated transcript start sites), enhancer regions (exemplified by p300 ChIP-Seq from embryonic forebrain, midbrain, limb and embryonic stem cells), CpG islands and intragenic regions were generally more unique that the intergenic region (Fig. 5a). We also looked at the uniqueness pattern within gene regions, and found introns to contain slightly less unique sequence while UTRs had highest uniqueness (Fig. 5b). From these analyses we note that in general, only a small increase in uniqueness is observed when increasing read lengths above 50 nt, and for particular regions such as CpG islands the increase in uniqueness levels off already at 25 nt. Therefore, analyses of regulatory regions do not require particular long reads, but can benefit from using uniqueness normalization in the peak calling as shown for ChIP-Seq data [9].

Bisulfite treatment of DNA converts unmethylated cytosines to uracil and is widely used to map DNA methylation levels with single nucleotide resolution. To achieve an unbiased mapping of bisulfite sequencing reads to the genome, one first converts all cytosines to thymidines in the reads and then aligns them to a genome index generated from fully converted chromosomes (effectively only containing A, G and T bases). We next investigated how bisulfite treatment affected mappability, and generated similar MULTo files for mouse and human bisulfite converted genomes (see Methods). As expected, the mappability is generally lower in the bisulfite-converted genome. However, for reads longer than 35 nt, the difference in mappability between the bisulfite and normal genome is quite low (Fig. 5c–d), consistent with a recent study on bisulfite sequencing [14]. Since DNA methylation of regulatory regions (enhancers, promoters and CpG islands) affects gene expression [15], [16], these regions are of special interest when performing bisulfite sequencing. We found that these regulatory regions are even less affected by loss of mappability than intragenic or intergenic regions, or the genome as a whole (Fig. 5c). We conclude that when using read lengths of 35 or above, bisulfite conversion causes only a small decrease in mappability

## Discussion

RNA-Seq is becoming the gold standard for genome-wide transcriptome analyses. When estimating absolute expression levels within a sample, it becomes important that the length normalization is faithfully recapitulating both the length of the expressed transcript isoform [17] and that one carefully compensates for uniqueness or multimapping reads. We demonstrated that discarding multimapping reads and correctly adjusting the transcript length to only consider uniquely mappable positions achieved the most controlled compensation for uniqueness. These results were in agreement with a recent comparison of RNA-Seq derived expression levels with qRT-PCR data, where they found that correction for uniquely mappable positions improves expression level estimations [5]. In the other methods, it is possible that transcripts with low proportion of unique positions will obtain too low raw RPKM and therefore have lower probabilities of obtaining multimapping reads with the consequence of a final underestimation of their expression levels. Although these analyses focused on gene and transcript expression estimation, it is clear that the uniqueness compensation will be equally important for analyses of parts of transcripts, e.g. in exon inclusion levels [12]. Indeed, the importance of correct uniqueness compensation should increase when estimating absolute expression or inclusion of short transcript parts. Our results on mappability of paired-end data as a function of read and fragment lengths are effects that are intrinsic to previous analyses, which also added inference of indirect isoform-specific information [12].

The uniqueness files we used throughout this study did not consider mismatches. Although read alignment procedures often allow for mismatches, only reads with a single “best” match are normally kept and reads that instead map equally well to multiple locations are discarded as multimapping reads. For example, Bowtie users generate these alignments using the “best” and “strata” flags. Using uniqueness files where each chromosomal coordinate has to be unique and more than a number of mismatches away from the rest of the genome would likely be of limited use since they are overly conservative and would lead to unnecessary loss of sequence information. Finally, considering the low error rates in base calling on commonly used sequencing platforms (∼1% on Illumina sequencers), it is unlikely that sequencing errors alone would cause reads to map to wrong locations.

Although the read length range used in these analyses has a maximum of 255 nts, the same procedure can easily be extended to include read lengths up to 65,536 nts by simply storing the minimum unique length for each position in two bytes instead of one.

There are however limitations in using a method that discard multimapping reads. First, the approach is not as effective in transcript reconstruction experiments, since non-unique regions will get no coverage that will cause fragmented reconstructions. Second, recently duplicated paralogues with no uniquely distinguishing positions will escape quantification. Therefore, we propose that different uniqueness compensation approaches would have to be used for expression level estimation and transcript reconstruction.

The creation of uniqueness files is more challenging for paired-end sequencing experiments, since the minimum unique length of a pair of reads is dependent upon the fragment length distribution. It is therefore not possible to get an absolute value of the minimum unique length for each position, which preclude us from creating genome-wide paired-end uniqueness files. Instead, paired-end uniqueness files can be generated for specific genomic regions and in this study we estimated the proportion of unique positions in each transcript, still over a range of read lengths but at different given mean fragments length distributions. This information is well suited to normalize paired-end RNA-Seq data for uniqueness.

There are other next-generation sequencing applications where it could be equally important to correct for the uniquely mappable positions. Lack of uniqueness could potentially affect the ability to identify peaks of reads in e.g. ChIP-Seq or DNAse-Seq applications. In general, we found a fairly high uniqueness in regulatory regions indicating that mappability normalization might be more important to avoid false positive peak calls than false negatives. We also reasoned that bisulfite-Seq might be more dependent upon uniqueness compensation due to the reduced complexity of the genome, but we found only minor decrease in mappability for fully converted reads. However, uniqueness compensation might be more important when comparing peak strengths in data sets generated with different read lengths.

Finally, we have made both scripts and MULTo files (for hg19 and mm9) available from our website (MULTo website. Available: http://sandberg.cmb.ki.se/multo, Accessed Dec 12 2012). This MULTo framework can be easily integrated into software to leverage the use of accurate uniqueness compensation for RNA-Seq and to eliminate the need to make uniqueness files for each new read length encountered.

## Materials and Methods

### Generation of MULTo files

To determine the minimum unique length (MUL) for each genomic position, we iteratively evaluate reads of different lengths from each genomic coordinate for uniqueness in the genome. Since we store the information of minimum read lengths in single bytes, the range of read lengths is limited by the numbers a byte can take on, i.e. 0–255. This range well covers the common read lengths used for sequencing today, and as default we query read lengths between 20 and 255. The program takes in a Fasta file for each chromosome, and constructs a temporary Fasta file with artificial reads from each genomic position which it then maps to the genome with Bowtie [18]. We typically process a block of 10 M reads at a time, and we make sure to only obtain uniquely mappable positions and allowing for no mismatches (−m 1 and –v 0 options in Bowtie). In the first round, we query uniqueness at the maximum read length (255 by default) to be able to discard everything that is multimapping at this length, and write zeros to the genomic positions of these reads to indicate that they are not mappable within the span of 20–255 nts. In the second round, we query genome uniqueness at the minimum read length as above, and all positions unique at this length can be left out from further queries and we write 20 at each coordinate in the binary MULTo files generated. To find the individual uniqueness of each genomic coordinate in the range 20–255, we incrementally step up in read length 25 nts at a time. In each such iteration, we first find the multimapping reads at this length, and those that where unique are queried for shorter read lengths, until the minimum required read length for unique mapping is found (see more details in Figure S2). This iterative procedure was run with multi-threading both for each Bowtie mapping (where we use 15 processors) and we run several chromosomes in parallel.

### Creating Bisulfite MULTo files

To create bisulfite MULTo files we had to make some modifications to the algorithm due to the fact that the two strands will no longer be reverse complementary when all the Cs are converted to Ts. Since Bowtie will by default map reads to both strands, we instead had to create bisulfite converted genome sequence for both the forward and reversed strand, and then build bowtie indexes for both. Due to size limitations for input files to the bowtie-index builder we had to create separate indexes for forward and reverse strands. The genomic reads are therefore mapped twice with bowtie for this application, once for each strand. A read is first mapped to the strand it came from, using option −m 1 and –norc in Bowtie to suppress reports of multimapping reads and reverse complementary mapping respectively. When the read is mapped to the other strand, we use option −k 1 instead of −m 1. In this way multimapping is allowed, but only the first valid alignment is reported to speed up the search. By removing all reads that pass through the second mapping from the ones from the first mapping we will only get the reads that were unique left. By default two bisulfite MULTo files are created; one for forward strand and one for reverse strand.

### Creating Transcriptome MULTo files

The transcript MULTo files were based on reference annotations (RefSeq by default). Reads are then created from these files by the same approach as for the genome. Spliced transcripts are generally around 1–3 kb in length, which means that every transcript will generate a few thousand artificial reads. We found that the speed of bowtie alignments for fewer reads was quite low. The mapping time per read does not reach a plateau until 10 million reads or more are aligned in batch (data not shown). Therefore we combined together around 10 million reads from different transcripts and run them together through the algorithm, only separating them at the step where minimum uniqueness read lengths are saved to an array for each transcript.

To find the uniqueness of transcripts we need to align reads both to the genome and to other transcripts, and we therefore create a bowtie-index that contains both the full genome and transcriptome. Since reads can often map to the exact same (genomic) positions in several transcripts as well as to the genome, we must allow multimapping in this approach (using the –a option in Bowtie). Allowing multimapping will slow Bowtie down significantly, and we therefore took measures to only perform the least possible multimappings. This is achieved by maximally allowing as many multimapping reads as the number of existing overlapping transcripts for a genomic locus in the reference annotation. We therefore used the flag −m for bowtie to only allow as many multimappings as the number of overlaps, plus one for the genomic hit, since if there are more we can be sure that this read was mapping to another region as well. The software package includes a function for creating transcript Fasta files which also determines the number of overlaps to other annotated genes and sorts them into folders according to the number of overlaps.

We further separated uniqueness into a gene level and transcript level uniqueness. For each multimapping read, we compared the genomic start and end positions of all matched locations to determine whether they were all derived from the same genomic locus. For transcript level uniqueness, only reads that map to exactly one start position **and** one end position are considered unique, while gene level MUL values only requires that all reads map to the same start **or** end position. The uniqueness of each transcript is stored in separate MULTo files.

### Creating paired-end uniqueness files

An absolute minimum unique length cannot be determined for paired-end data, due to differences in fragment length between and within samples. Instead we create uniqueness files with an estimation of the number of uniquely mappable positions in each transcript for a range of read lengths, given a fragment length distribution. MULTo assumes a normal distribution of fragment lengths, where the mean and standard deviation is given as input (default values are mean = 250, std = 25). Paired-end fragments are simulated by drawing a number (5 by default) of random lengths from the given Gaussian distribution for each position in the transcript. Fragments with an end position outside the transcript will be discarded. Mate pairs of different lengths are created from these fragments and mapped to the genome by the same algorithms as gene-level and transcript-level uniqueness queries, but instead of saving a minimum unique length for each position, the proportion of the fragments that are uniquely mapped at each step is saved. When running bowtie, the minimum (−I) and maximum (−X) insert size is given as mean ±3 standard deviations, which should cover 99.7% of all fragments in the Gaussian distribution. For the default case this will be −I 175 and −X 325. An estimate of the unique length of the transcript at each read length is then calculated as transcript length • (uniquely mapped fragments/all fragments) and this is presented in the output in a table format. Observe that read lengths close to the mean fragment length is not very useful in this approach; since bowtie discards mappings where mate1 and mate2 completely overlap.

### Alignment of RNA-Seq reads

We analyzed mixed-tissue data from the Illumina Human Body Atlas 2.0 Project (GSE30611) that were sequenced for 100 bp single-end, stranded reads. The raw reads in fastq files were iteratively trimmed down to shorter read lengths to analyze the effect of read length on mappability and expression level estimation. Reads were aligned using TopHat against respective genome assembly (hg19 and mm9) and raw junctions derived from RefSeq annotations were provided. We initially allowed multimapping (using –g 255) and two mismatches (−n 2) and then identify the “best” match with the fewest number of mismatches. Reads with only one best matching locations were considered unique. The minimum anchor length for junction-spanning reads was set to 4 nt.

### Gene expression level estimation

Using rpkmforgenes, we generated RPKM expression levels using only alignments files with uniquely mappable reads and corrected the transcript lengths using a MULTo file with gene level uniqueness superimposed upon the genomic uniqueness information. The parameters used were: “-rmnameoverlap (to ignore exons shared by multiple genes), -allmapnorm (to normalize the depth against all mapped reads), -fulltranscript (to not remove UTRs), -samse (fast option for unique SAM format, single-end, with only uniquely mappable reads)”. Expression level estimations using cufflinks were based on alignment files with multimapping read (however redundant mappings from trancriptomes and genomes were removed). We used the –multi-read-correct flag in cufflinks to get best possible weighting of reads mapping to multiple locations. Expression levels for mouse tissues that were estimated using ERANGE were downloaded [2]. We re-analyzed their raw data (also trimming lengths to 25 nts) to generate uniquely mapping reads for gene expression analyses in rpkmforgenes [13].

### Gene and genomic region annotations

The gene annotations used for mappability profiles over transcripts were based on RefSeq transcripts for mm9 assembly downloaded from UCSC Genome Browser on July 3^rd^ 2011. The CpG island annotations were downloaded from UCSC Genome Browser mm9 annotation track (30 August 2011). Cytoband annotations were downloaded from dChip website (http://sites.google.com/site/dchipsoft/home) on September 6^th^ 2011.

## Supporting Information

Figure S1 Schematic of the algorithm for finding minimum unique length. (**A**) 255 nt reads are created from the 10 million first positions, and mapped to the genome. Those positions that did not have a uniquely mapping read will be represented by a zero in an array, and later in the MUL-file. (**B**) The unique positions at 255 are further mapped at 20 nt. Those unique at 20 nt will be represented by 20 in the array, and the non-unique will be mapped at 45 nt (**C**). The reads that are non-unique at this step will iteratively be mapped at 25 nt higher until uniqueness is found. When uniqueness is found at this step, we know an upper limit where the position is unique (kHigh), and a lower limit where it is not unique (kLow). (**D**) We now go through the lengths between kLow and kHigh to find the exact length at which the position becomes unique. When the MUL value is found for all 10 million positions, the array is written to a binary file and the next block of 10 million positions is queried.
(TIF)

Figure S2 Creating transcriptome MUL files. The spliced transcript sequence is fetched from the genomic sequence into new Fasta files, from which Fasta files with artificial reads are created for mapping against the genome and transcriptome. A read is considered unique at “gene level” if it maps to only one genomic locus (same start or end position), while it is considered unique at “transcript level” only when it maps to only one transcript.
(TIF)

Figure S3 Uniqueness at single read and two lengths of paired-end fragments. (**A**) Proportion unique positions from all transcripts at gene-level. (**B**) Proportion unique positions from all multi-isoform genes at the transcript-level.
(TIF)

Figure S4 Comparisons between raw RPKM values. (**A**) Cufflinks RPKM values from only unique reads are in general similar to our raw RPKM values, except for a subset with higher RPKM in Cufflinks. (**B**) ERANGE raw values were more consistently similar to our raw values. (**C**) The subset of higher RPKMs in Cufflinks was due to Cufflinks overestimating the expression of short transcripts. (**D**) Comparison of cufflinks result when allowing a maximum of 255 or 20 multi hits.
(TIF)
