# Supplementary figures and images for: Efficient and Comprehensive Representation of Uniqueness for Next-Generation Sequencing by Minimum Unique Length Analyses
Source: PLoS One. 2013 Jan 18;8(1):e53822. doi: 10.1371/journal.pone.0053822 (PMC3548888; doi:10.1371/journal.pone.0053822)

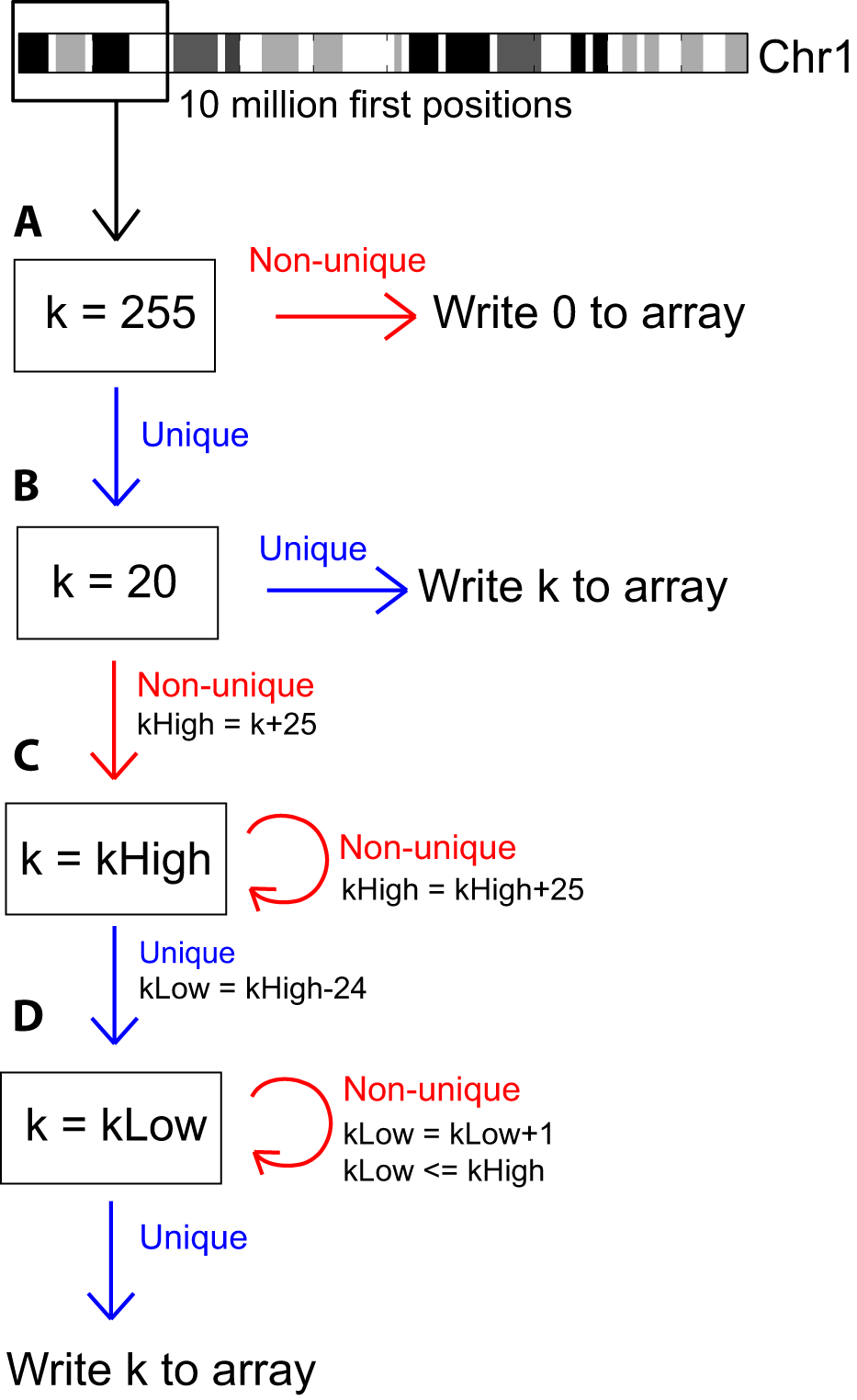

Supplement: Figure S1 — Schematic of the algorithm for finding minimum unique length. (A) 255 nt reads are created from the 10 million first positions, and mapped to the genome. Those positions that did not have a uniquely mapping read will be represented by a zero in an array, and later in the MUL-file. (B) The unique positions at 255 are further mapped at 20 nt. Those unique at 20 nt will be represented by 20 in the array, and the non-unique will be mapped at 45 nt (C). The reads that are non-unique at this step will iteratively be mapped at 25 nt higher until uniqueness is found. When uniqueness is found at this step, we know an upper limit where the position is unique (kHigh), and a lower limit where it is not unique (kLow). (D) We now go through the lengths between kLow and kHigh to find the exact length at which the position becomes unique. When the MUL value is found for all 10 million positions, the array is written to a binary file and the next block of 10 million positions is queried. (TIF) [file pone.0053822.s001.tif]

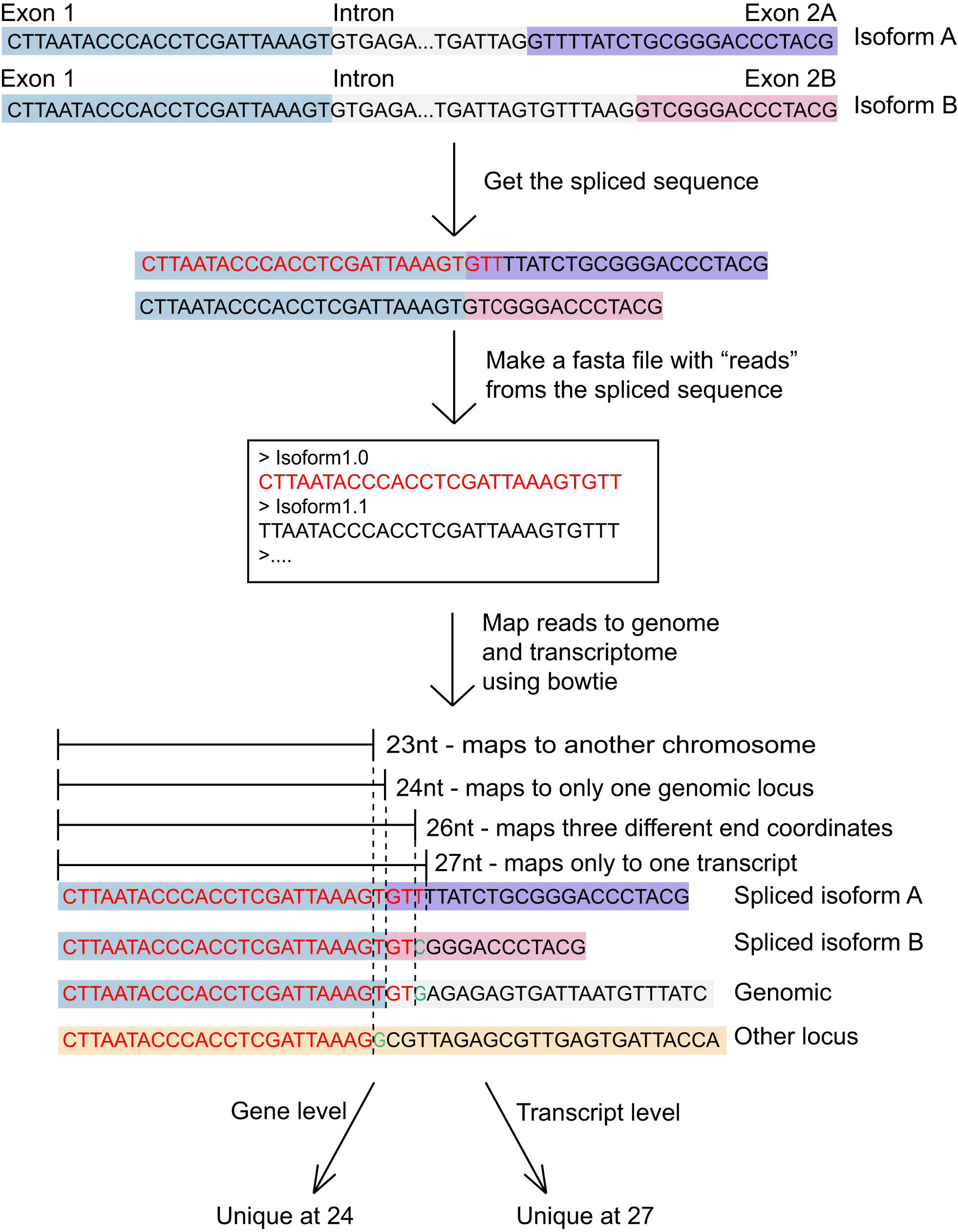

Supplement: Figure S2 — Creating transcriptome MUL files. The spliced transcript sequence is fetched from the genomic sequence into new Fasta files, from which Fasta files with artificial reads are created for mapping against the genome and transcriptome. A read is considered unique at “gene level” if it maps to only one genomic locus (same start or end position), while it is considered unique at “transcript level” only when it maps to only one transcript. (TIF) [file pone.0053822.s002.tif]

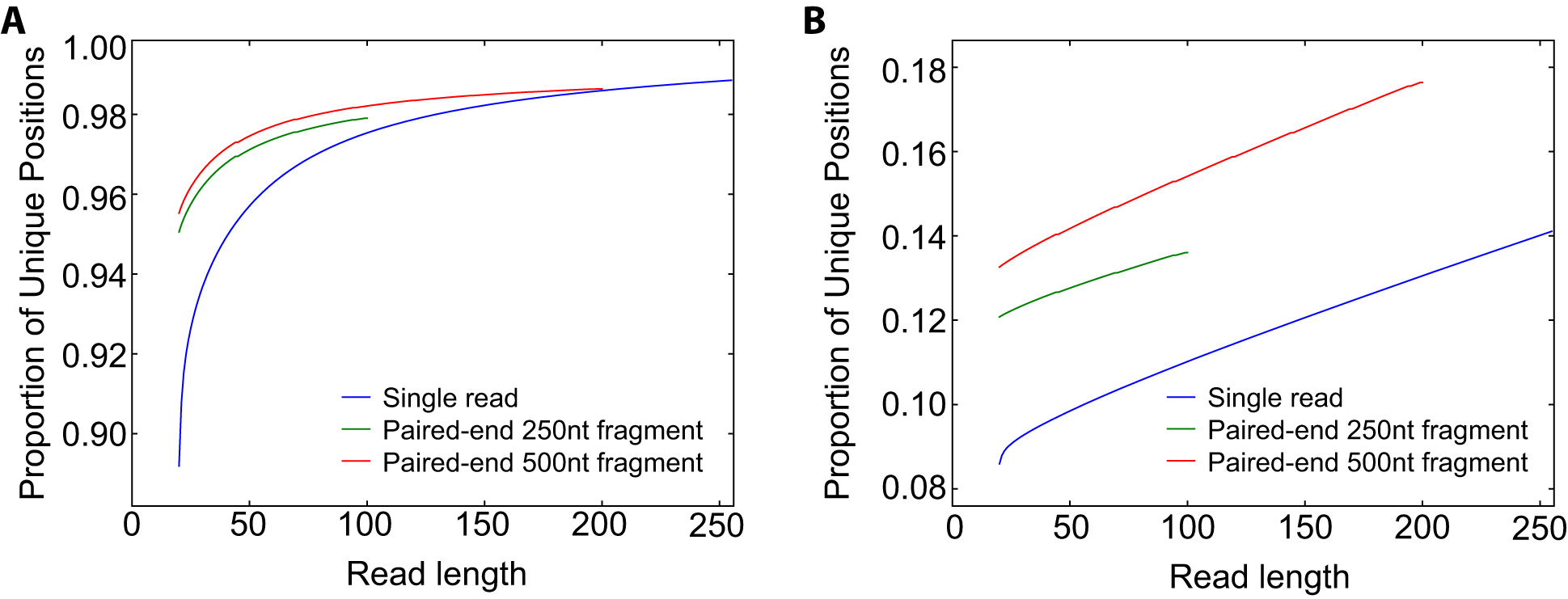

Supplement: Figure S3 — Uniqueness at single read and two lengths of paired-end fragments. (A) Proportion unique positions from all transcripts at gene-level. (B) Proportion unique positions from all multi-isoform genes at the transcript-level. (TIF) [file pone.0053822.s003.tif]

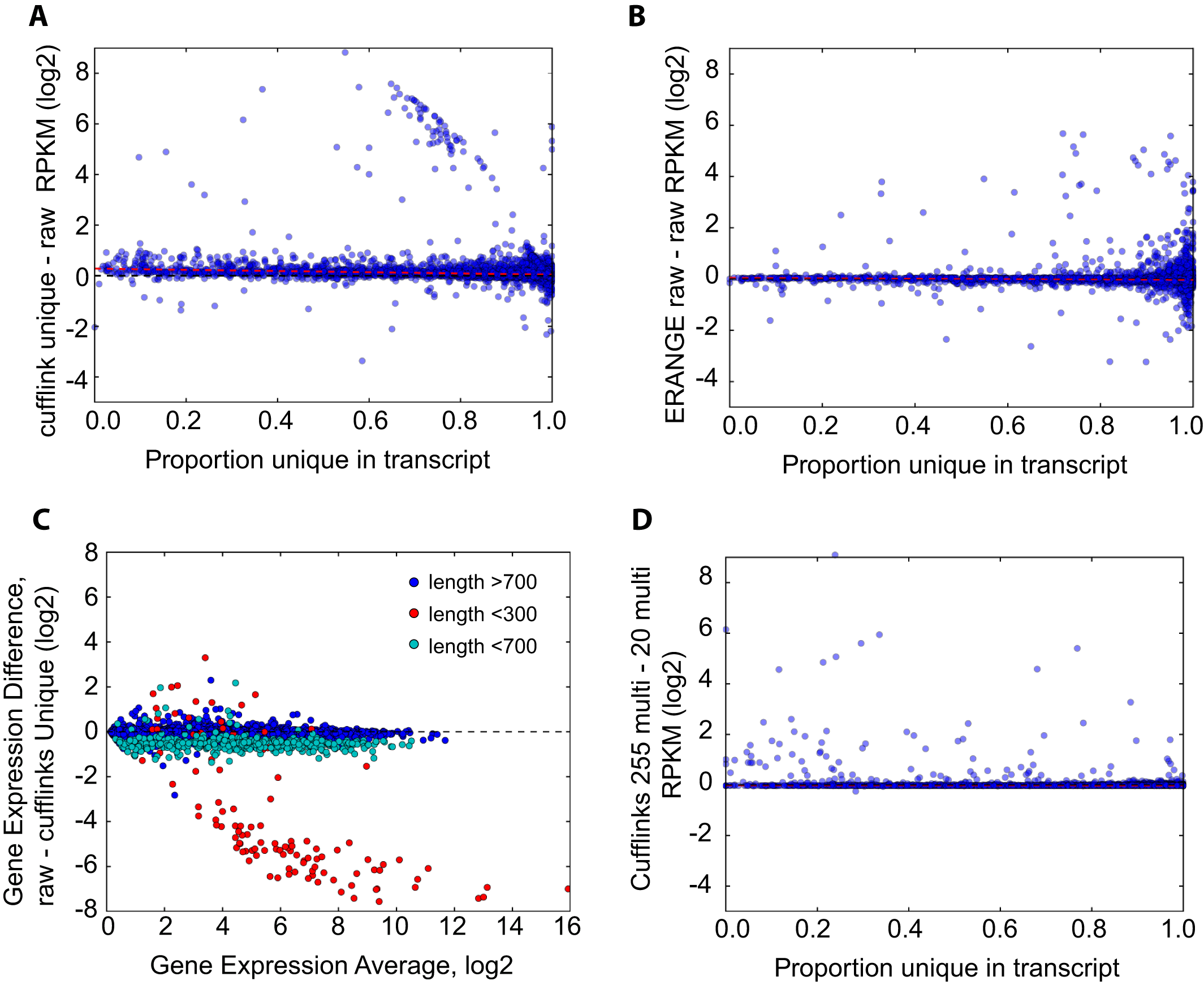

Supplement: Figure S4 — Comparisons between raw RPKM values. (A) Cufflinks RPKM values from only unique reads are in general similar to our raw RPKM values, except for a subset with higher RPKM in Cufflinks. (B) ERANGE raw values were more consistently similar to our raw values. (C) The subset of higher RPKMs in Cufflinks was due to Cufflinks overestimating the expression of short transcripts. (D) Comparison of cufflinks result when allowing a maximum of 255 or 20 multi hits. (TIF) [file pone.0053822.s004.tif]
